# Fabrication of High-resolution Graphene-based Flexible Electronics via Polymer Casting

**DOI:** 10.1038/s41598-019-46978-z

**Published:** 2019-07-22

**Authors:** Metin Uz, Kyle Jackson, Maxsam S. Donta, Juhyung Jung, Matthew T. Lentner, John A. Hondred, Jonathan C. Claussen, Surya K. Mallapragada

**Affiliations:** 1 Department of Chemical and Biological Engineering, Iowa State University, Ames, Iowa 50011 USA; 2 Department of Mechanical Engineering, Iowa State University, Ames, Iowa 50011 USA

**Keywords:** Nanoscience and technology, Electronic devices

## Abstract

In this study, a novel method based on the transfer of graphene patterns from a rigid or flexible substrate onto a polymeric film surface via solvent casting was developed. The method involves the creation of predetermined graphene patterns on the substrate, casting a polymer solution, and directly transferring the graphene patterns from the substrate to the surface of the target polymer film via a peeling-off method. The feature sizes of the graphene patterns on the final film can vary from a few micrometers (as low as 5 µm) to few millimeters range. This process, applied at room temperature, eliminates the need for harsh post-processing techniques and enables creation of conductive graphene circuits (sheet resistance: ~0.2 kΩ/sq) with high stability (stable after 100 bending and 24 h washing cycles) on various polymeric flexible substrates. Moreover, this approach allows precise control of the substrate properties such as composition, biodegradability, 3D microstructure, pore size, porosity and mechanical properties using different film formation techniques. This approach can also be used to fabricate flexible biointerfaces to control stem cell behavior, such as differentiation and alignment. Overall, this promising approach provides a facile and low-cost method for the fabrication of flexible and stretchable electronic circuits.

## Introduction

The field of flexible and stretchable electronics has garnered increasing interest due to versatility for unique on-body applications including but not limited to portable energy-harvesting devices, electronic skin, wearable electronic devices, and sensors^1^. Graphene has recently received a lot of interest as a promising conductive material for flexible electronics device fabrication due to its exceptional electrical, optical and mechanical properties^2–4^. Graphene has been used in combination with various flexible polymer substrates (e.g., polyethylene terephthalate (PET), polyimide (PI), and polydimethylsiloxane (PDMS))^5–10^ as well as other non-conventional substrates (e.g., paper, tape, and cloth)^11–14^ through different fabrication processes for the development of next-generation flexible electronics^15–21^. The flexible nature of these substrates enables protection of graphene electrodes from various deformations, and facilitates their installation onto irregular, curvilinear surfaces^4,22^.

Conventional methods such as chemical vapor deposition (CVD) can fabricate low-cost and large-scale graphene films on metal substrates at high growth temperatures (300–1000 °C), and the graphene is subsequently transferred to the substrate of interest^23–27^. Polymer-based transfer methods, that serve as an intermediate step between the metal substrate and the final target substrate for the graphene, involve mostly poly(methyl methacrylate) (PMMA) and polydimethylsiloxane (PDMS) along with others such as PET, PI and polyvinyl alcohol (PVA), due to their surface energy and adhesion forces between polymer/graphene and polymer/target substrate interfaces^23,28^. A major challenge for polymer-assisted graphene transfer is protecting the graphene integrity and preventing the mechanical deformation and destruction during transfer. Moreover, polymer-assisted graphene transfer requires substantial processing, such as stamping, plasma etching, chemical etching, washing, and high temperature baking^28^. Despite the optimization and enhancements in transfer processes^28^, there are still concerns regarding the remaining residues after the transfer, deteriorating the electronic properties of graphene^23^. Other than polymers, thermal release tape (TRT)-based graphene transfer has also been used for the roll-to-roll (R2R) technique; however, this method requires applied pressure, etching and high temperatures (~100 °C) to separate the tape from the graphene^23,28^.

Graphene-based flexible electronics can also be fabricated through various printing techniques such as inkjet printing, gravure printing, screen printing, and offset printing, providing high throughput large-scale production with low raw material consumption and reduced cost^29,30^. However, these techniques typically require additional postprocessing, particularly high temperature sintering, which limits the substrate selection to thermally sensitive substrates, such as poly(ethylene naphthalate) (PEN), PI or PET possessing relatively low glass transition temperatures^29,30^. Graphene transfer after printing may be required for some cases, where mostly polymer-assisted transfer processes described earlier are used^29^.

Transfer of graphene via sticky/adhesive tape peeling^31,32^, transfer printing^33^, or micro transfer molding^34^ are other potential fabrication methods. Graphene transfer via patterned sticky/adhesive tape peeling has difficulties in controlling the graphene feature size and resolution^32^. Although recent studies demonstrated certain improvements in patterned graphene transfer via “Stick-and-Transfer” process, this method still requires use of high graphene amounts to coat PDMS negative pattern features, additional tape peeling for surface cleaning, and is only valid for adhesive tapes, limiting the use of polymer based substrates^22^. Transfer printing provides high-resolution patterning of graphene through transferring of graphene patterns from a patterned template or mold to a receiving substrate^33,35,36^. However, it requires etching, PDMS stamping and removal^37–39^, or DMSO-based surface energy modification of PDMS molds^40^. In micro transfer molding, the patterned surface of a stamp is placed on the target substrate and the channels are filled with conductive graphene solution through capillary action. This process then requires vacuum drying and removing the stamp from the final substrate^34,41^.

In summary, it is difficult to conduct the entire graphene-based flexible electronic device fabrication procedures such as CVD, printing and lithography, directly on the target flexible substrate due to the requirements of harsh chemical, physical or thermal treatments that can significantly deform the target polymeric substrates, particularly natural or synthetic biodegradable polymers possessing low thermal stability^42–44^. This situation not only limits the target substrate material selection but also the potential application areas, especially in the healthcare and biomedical fields^43,45^. Because of this, most polymer-based transfer methods are an intermediate step between the donor and receiver substrates and use sacrificial polymer carrier layers mostly limited by PMMA or PDMS stamping^46–48^, etching^48,49^, hot lamination/delamination^38,49,50^, or electrochemical bubbling^51^ to transfer the graphene patterns to the target substrate. Recent studies have demonstrated direct transfer of graphene on PET substrate based on selective dewetting; however, this method requires UV curable adhesive^35,52^. Similarly, CVD grown graphene was also transferred to PVA through drop casting and lamination^53^ and to PDMS surface via drop casting and peeling approach with low transfer efficiency^54^. There are some other studies reporting the direct transfer of conductive silver using polymer casting^55^; however, transfer of graphene via simple polymer casting directly to the target polymeric substrate could be an alternative, simple, fast, green and cost-effective approach allowing the use of variety of flexible substrate materials and eliminating the aforementioned processing steps.

Here we propose a facile and versatile graphene transfer method at room temperature based on simple polymer casting that does not require thermal processing, etching, stamping or UV treatment. This process is simply based on the differences in the surface energies and adhesive forces between the graphene/mold and graphene/target polymer substrate created during the polymeric film formation^23,28^. With this approach, we are able to fabricate high-resolution, small feature sized (as small as 5 µm; 15 µm width and 5 µm depth) conductive graphene patterns/circuits (sheet resistance of ~0.2 kΩ/sq without the need for post-deposition annealing) on various flexible polymeric substrates. Briefly, our method consists of two main steps; (i) the formation of graphene patterns/films on substrates/molds via conventional methods such as CVD, channel filling or ink-jet printing and (ii) direct casting of target substrate polymer solution on the graphene patterned substrates/molds and direct transfer of graphene patterns to the target substrate via peeling off upon drying and film formation. This makes our method versatile allowing the use of different polymers including natural/synthetic, biodegradable/non-biodegradable polymers (such as Poly-L-Lactic Acid (PLLA), Cellulose Acetate (CA), Gelatin (GEL), Poly Lactic-co-Glycolic Acid (PLGA) or Whey Protein Isolate (WPI)) with well-defined characteristics and provides precise control of 3D microstructural and mechanical properties (such as film porosity, pore size, elasticity etc.) of the target substrate material with high resolution graphene patterns (feature dimensions of ~5 µm width/depth). This process also enables graphene-based circuit design on biodegradable polymeric films which is not possible with chemically or thermally degrading, lithographic patterning techniques. Our method also requires the use of lithography only for the preparation of molds with high resolution and small feature sized patterns; however, it needs to be done only once. On a broader scale, the use of this new room-temperature facile method to fabricate biodegradable, biocompatible, flexible, and electrically-conductive graphene circuits could pave the way for various applications including tissue engineering, robotics, implantable heart sensors, brain-computer interfaces, or low-cost wearable sensors^56–58^.

## Results and Discussion

**Figure 1:**
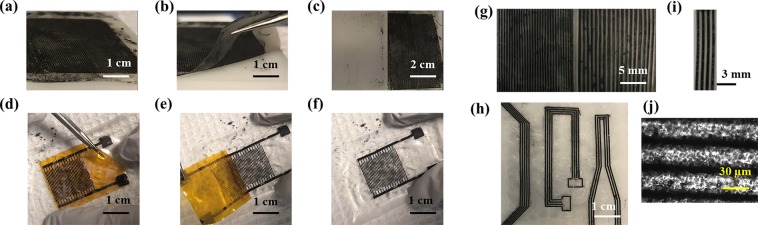
Graphene circuits fabricated via polymer casting. (**a**–**c**) Images showing the steps of graphene transfer from rigid Delrin mold via Poly-L-Lactic Acid (PLLA) casting. (**a**) Casting of PLLA solution on graphene patterns formed on Delrin surface via microfluidic filling. (**b**) Peeling off the PLLA film from the rigid Delrin surface and transfer of graphene upon drying of polymer solution and film formation. (**c**) PLLA film with graphene patterns on the surface and Delrin substrate after the process. (**d**–**f**) Images showing the steps of ink-jet printed graphene transfer from flexible polyimide substrate via Cellulose Acetate (CA) casting. (**d**) Casting of CA solution on graphene patterns printed on flexible polyimide substrate. (**e**) Peeling off the CA film from the polyimide substrate and transfer of graphene upon drying of polymer solution and film formation. (**f**) CA film with graphene patterns on the surface and polyimide substrate after the process. Graphene pattern of (**g**) 400 µm and 200 µm width, 150 µm depth on Gelatin (GEL) film; (**h**) 300 µm width, 150 µm depth on Poly Lactic-co-Glycolic Acid (PLGA) film; (**i**) 400 µm width, 150 µm depth on Whey Protein Isolate (WPI) film; (**j**) 15 µm width, 50 µm depth on PLLA film (image take by light microscope).

The developed method is focused on direct transfer of graphene-based patterns from rigid or flexible substrates to the polymeric flexible films via polymer casting. The method consists of three main steps; (i) Preparation of graphene-based patterns/films via channel filling, ink-jet printing or CVD on rigid or flexible substrates/molds; (ii) Casting of the target substrate polymer solution on the graphene-based patterns/films formed on substrates/molds; (iii) Drying of the solvent and formation of films followed by peeling off the films from the substrate/mold, transferring the graphene pattern from substrate/mold surface to the target polymeric film surface. The application steps of the graphene transfer via direct polymer casting on rigid Delrin and ink-jet printed flexible polyimide substrate was shown in Fig. 1a–f, respectively. As seen in the figures, a complete transfer of graphene was obtained for both cases. In addition, this process works for different polymers and their respective solvents (Fig. 1a–f).

This method is also versatile and can be applied to many different polymeric materials including but not limited to PLLA, PLGA, CA, GEL and WPI films (Fig. 1g–j). It was noted that almost 100% of the graphene patterns present on the substrates were successfully transferred to the polymeric film surface (Fig. 1). This high transfer efficacy could be mostly attributed to the surface properties, particularly the hydrophobicity of the substrate material. The hydrophobic substrates, such as Delrin, Teflon or polyimide, make it easy to remove the formed films along with the graphene pattern transfer. It was observed that this process does not depend on the polarity or viscosity of the polymer solution since the natural or synthetic polymers dissolved in polar or non-polar solvents. For instance, 10% PLLA dissolves in chloroform, which is a non-polar solvent, while 5% GEL or WPI dissolve in water, which is a polar solvent. For both cases we observed 100% of graphene transfer. Practically, the polymer casting-based graphene patterning and transfer technique utilizes adhesion forces of two contacting materials at the interface and their respective surface energies^22^. The difference in the surface energies between the polymer solution and graphene patterns upon the formation of polymeric films makes it possible to remove the graphene pattern precisely from the solid mold surface and transfer the patterned graphene onto the polymeric film surface. The work of adhesion at the graphene-polymer interface is higher than the work of adhesion at the graphene-mold interface, which makes the complete transfer of graphene possible as described in Equation 1^22^

1 $${W}_{A-B}=4\,(\frac{{\gamma }_{A}^{d}\,{\gamma }_{B}^{d}}{{\gamma }_{B}^{d}+{\gamma }_{B}^{d}}+\frac{{\gamma }_{A}^{p}\,{\gamma }_{B}^{p}}{{\gamma }_{B}^{p}+{\gamma }_{B}^{p}})$$

where γ^d^ and γ^p^ correspond to the dispersion and polar components of surface energy (γ = γ^d^ + γ^p^). The theoretically calculated work of adhesion between each material interface support the proposed hypothesis. For instance, W_Graphene-Delrin_ (77 mJ/m^2^) < W_Graphene-PLLA_ (90 mJ/m^2^); W_Graphene-Delrin_ (77 mJ/m^2^) < W_Graphene-PLGA_ (88 mJ/m^2^); and W_Graphene-Delrin_ (77 mJ/m^2^) < W_Graphene-GEL_ (88 mJ/m^2^).

**Figure 2:**
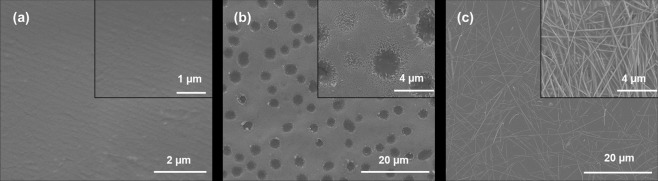
CVD grown graphene film transferred to a PLLA surface from (**a**) copper foil substrate and (**b**) quartz substrate. (**c**) CVD grown graphene-silver nanowire film transferred to PLLA surface from quartz substrate.

This graphene transfer approach can also be used to transfer the graphene patterns from one flexible substrate to another. For instance, we already demonstrated that the graphene patterns, ink-jet printed and post-processed (laser or thermal annealed), can easily be transferred to 3D microstructured and porous CA-based films using polymer casting approach (Fig. 1d–f). The successful transfer of graphene can also be supported by the calculated work of adhesion at the interface of graphene-polyimide and graphene-CA, W_Graphene-Polyimide_ (86 mJ/m^2^) < W_Graphene-CA_ (94 mJ/m^2^). Therefore, this fabrication approach allows formation of high-resolution patterns on the surfaces of versatile polymeric films as long as their free surface energies are sufficiently different to enable strong adhesion to one another. Similarly, we also demonstrated the potential of the direct polymer casting and peeling approach for the transfer of CVD grown graphene as illustrated in Fig. 2. The graphene films grown on copper foil (W_Graphene-Cu_ = 46 mJ/m^2^) (Fig. 2a) and quartz (Fig. 2b) as well as graphene-silver nanowire grown on quartz (W_Graphene-Quartz_ = 66 mJ/m^2^) (Fig. 2c) substrates via CVD approach were successfully transferred to the PLLA polymer surface (W_Graphene-PLLA_ = 90 mJ/m^2^) using our direct polymer casting approach. After the transfer, we did not observe a significant change in the resistance of graphene films, which indicates the efficiency of the transfer.

The conductivity of graphene was enhanced by applying thermal annealing (pre-annealing temperature of 75 °C for 3 h for 60 mg/mL concentration) prior to the application of graphene solution to create the substrate patterns via the channel filling approach. The transferred graphene via polymer casting has the sheet resistance of ~0.2 kΩ/sq. Similarly, the ink-jet printed and laser annealed graphene was also transferred from flexible polyimide substrate to CA substrate, which has the sheet resistance of ~0.7 kΩ/sq^59^.

**Figure 3:**
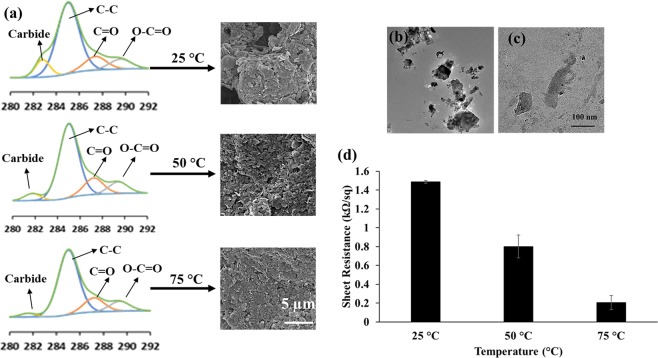
(**a**) XPS analysis of PLLA films with graphene surface patterns pre-annealed at 25, 50 and 75 °C and their corresponding structures as SEM images. TEM images of GNPs (**b**) non-annealed and (**c**) pre-annealed at 75 °C. (**d**) The change in sheet resistance with respect to pre-annealing temperature. According to the ANOVA analysis by Tukey’s method with a 95% confidence interval the p value was found to be smaller than 0.05 showing significant difference between the groups (p < 0.05). (n = 3, and error bar represents one standard deviation from the average).

It is possible to control the conductivity by changing the pre-annealing temperature along with the amount of graphene used for the filling approach. The effect of pre-annealing temperature on the graphene structure can also be observed via XPS analysis (Fig. 3a). The XPS analysis of graphene pattern on the PLLA film surface revealed the presence of classical C-C (~284.5 eV), C=O (~287.8 eV) and O–C=O (~288.9 eV) graphene peaks^60,61^ along with additional peaks around 282.7 eV, which potentially stems from the existence of graphene layers on the sample (Fig. 3a). With the increase in pre-annealing temperature from 25 °C to 75 °C, we did not notice a significant change in C-C (~284.5 eV), C=O (~287.8 eV) and O–C=O (~288.9 eV) graphene peaks, whereas a significant decrease in 282.7 eV peak, accompanied by a slight shift toward ~281.5 eV, was also observed, indicating the structural change upon pre-annealing. These peaks formed at binding energies around 282 eV are not classical graphene peaks, which generally can be observed in the range of 284–288 eV^60^, but could stem from the carbides in the structure of graphene^62^. Carbides are also known as excellent semiconductors and simple thermal annealing induces an *in-situ* transformation of silicon carbide films into the graphene matrix^62–65^. Therefore, the decrease in the carbide peak upon temperature annealing could be another reason for enhanced conductivity. In addition, we also observed formation of a dense and compact graphene structure as the pre-annealing temperature increases to 75 °C (Fig. 3a). The reduction in the GNP size and increase in surface area after pre-annealing and probe sonication, observed in TEM images (Fig. 3b,c), could also be another reason for enhanced electrical conductivity due to the continuous and densely packed graphene platelets within the film microchannels. In Fig. 3b, the GNP structure can be observed (non-annealed), while the structure of graphene was changed from nanoplatelet form to small particulate form upon pre-annealing and sonication (Fig. 3c). The change in the GNP size upon annealing and sonication was also confirmed by dynamic light scattering measurements. The non-annealed GNP size was around ~600 nm while upon annealing and sonication it became ~250 nm. The result of these structural changes is also reflected in the conductivity of the formed patterns. The increase in pre-annealing temperature resulted in a significant decrease in the sheet resistance of graphene patterns (Fig. 3d).

**Figure 4:**
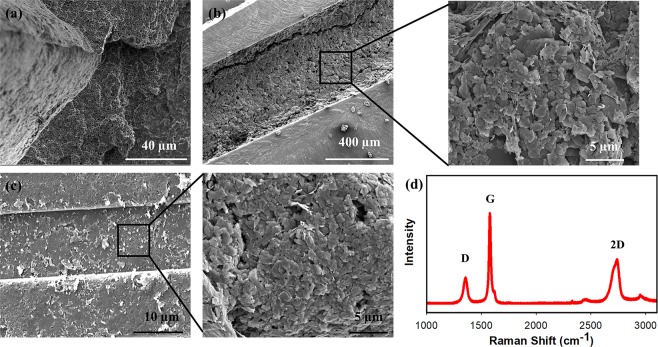
(**a**–**c**) SEM images of PLLA films and graphene micropatterns. (**a**) Cross section image of 3D porous microstructure of PLLA film. (**b**) Graphene micropatterns (500 µm width and 200 µm depth) on PLLA film surface. (**c**) Graphene micropatterns (15 µm width and 5 µm depth) on PLLA film surface. (**d**) Raman spectra of the transferred graphene on PLLA films.

The dense, compact and continuous filling of graphene patterns on the PLLA film surface are shown in Fig. 4. The 3D porous microstructure of substrate material was observed using dry-phase inversion technique on PLLA film (Fig. 4a). Using polymer casting method, it is possible to precisely control the microstructure of variety of substrate material, including but not limited with natural/synthetic or biodegradable/non-biodegradable polymers, which is not possible to obtain with currently available flexible electronic fabrication techniques. This demonstrate the versatility of our approach. Figure 4b demonstrates the graphene pattern with 500 µm of width and 200 of µm depth along with the structure of graphene on the PLLA film surface. In addition to this, it is also possible to obtain graphene patterns with small feature size. As shown in Fig. 4c, we were able to obtain graphene patterns on PLLA film surface with as low as 15 µm of width and 5 µm of depth. Although we have demonstrated examples mostly based on PLLA, it should be noted that this method is universal and can be applied to any type of polymer. Figure 4d displays Raman spectra for the transferred graphene on the PLLA film surface. The distinct and classical D, G, and 2D peaks (~1350, 1580, and 2700 cm^−1^, respectively) was observed^59,66^. Samples show a small D peak associated with lattice structure imperfections and edge plane defects in the graphene, as well as large G/2D peaks characteristic of sp2-hybridizated carbon (graphite/graphene structure)^59^. Graphene patterns displayed low (I_G_/I_D_) ratio (0.33 ± 0.01), which demonstrates that the transferred material is most adequately characterized as a multi-layer graphene structure^67–69^.

**Figure 5:**
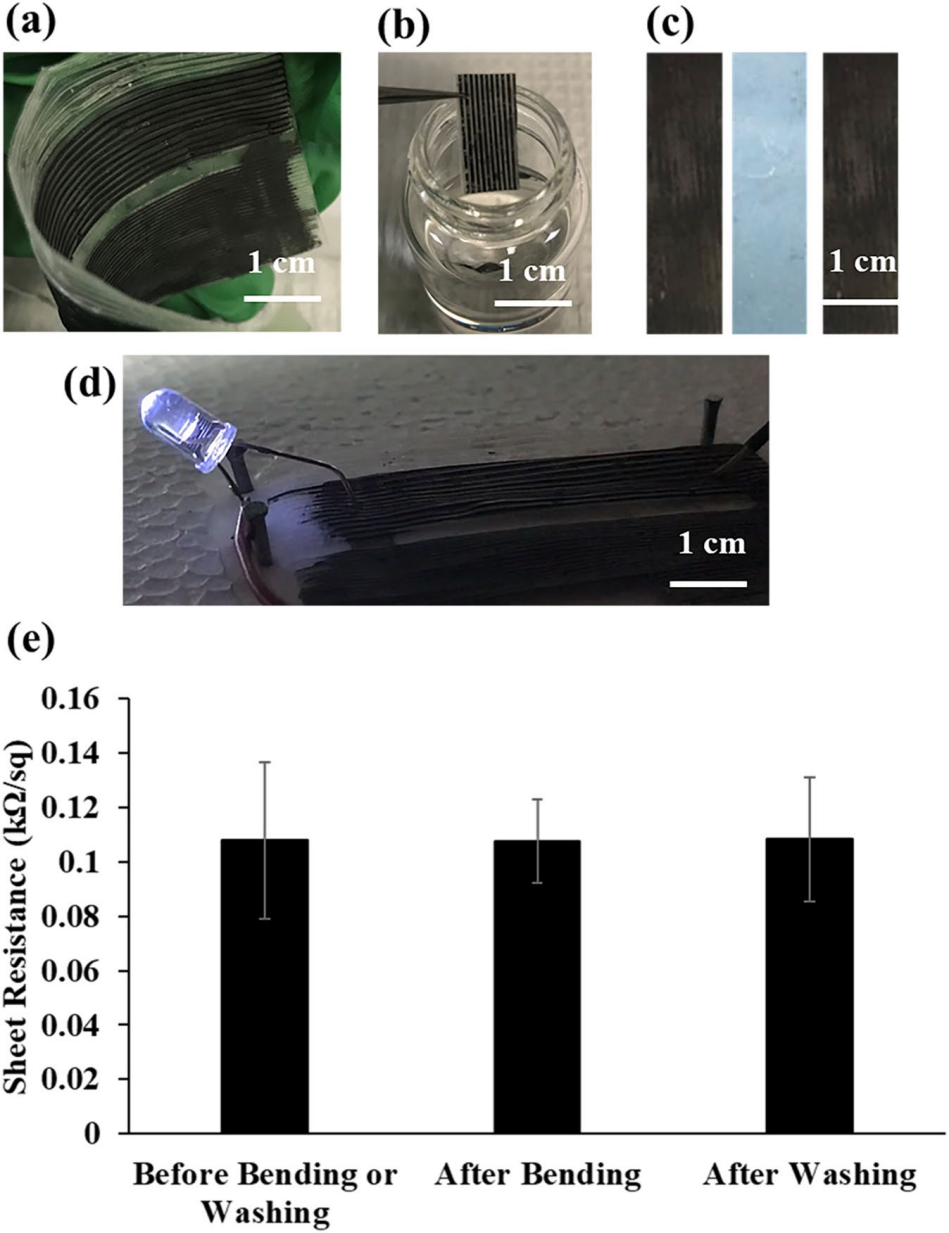
Stability and conductivity of the graphene patterns. Graphene patterns are flexible and bendable and keep their mechanical stability after (**a**) multiple bending (100 times), (**b**) washing cycles (24 h incubation) and (**c**) mechanical peeling applied via adhesive tape. (**d**) Graphene patterns have good conductivity and low resistance to build an active circuit. (**e**) Sheet resistance did not change after multiple bending and washing cycles. According to the ANOVA analysis by Tukey’s method with a 95% confidence interval the p value was found to be higher than 0.05 showing insignificant difference (p > 0.05). (n = 3, and error bar represents one standard deviation from the average).

The obtained graphene patterns showed significant stability after multiple bending and washing cycles (Fig. 5a,b). In addition, their assembly on the film surface is mechanically strong, and even after multiple stick and peel cycles using a commercially available adhesive tape, the graphene patterns were stable (Fig. 5c). Therefore, the graphene patterns obtained with polymer casting approach exhibited good stability and conductivity to obtain active circuits (Fig. 5d). These circuits boards were made of graphene patterns of 300 µm width and 100 µm depth connected to a 9V battery through copper wiring to light up an LED. In addition, they maintained their conductivity as the sheet resistance did not change after multiple washing and bending cycles (Fig. 5e).

**Figure 6:**
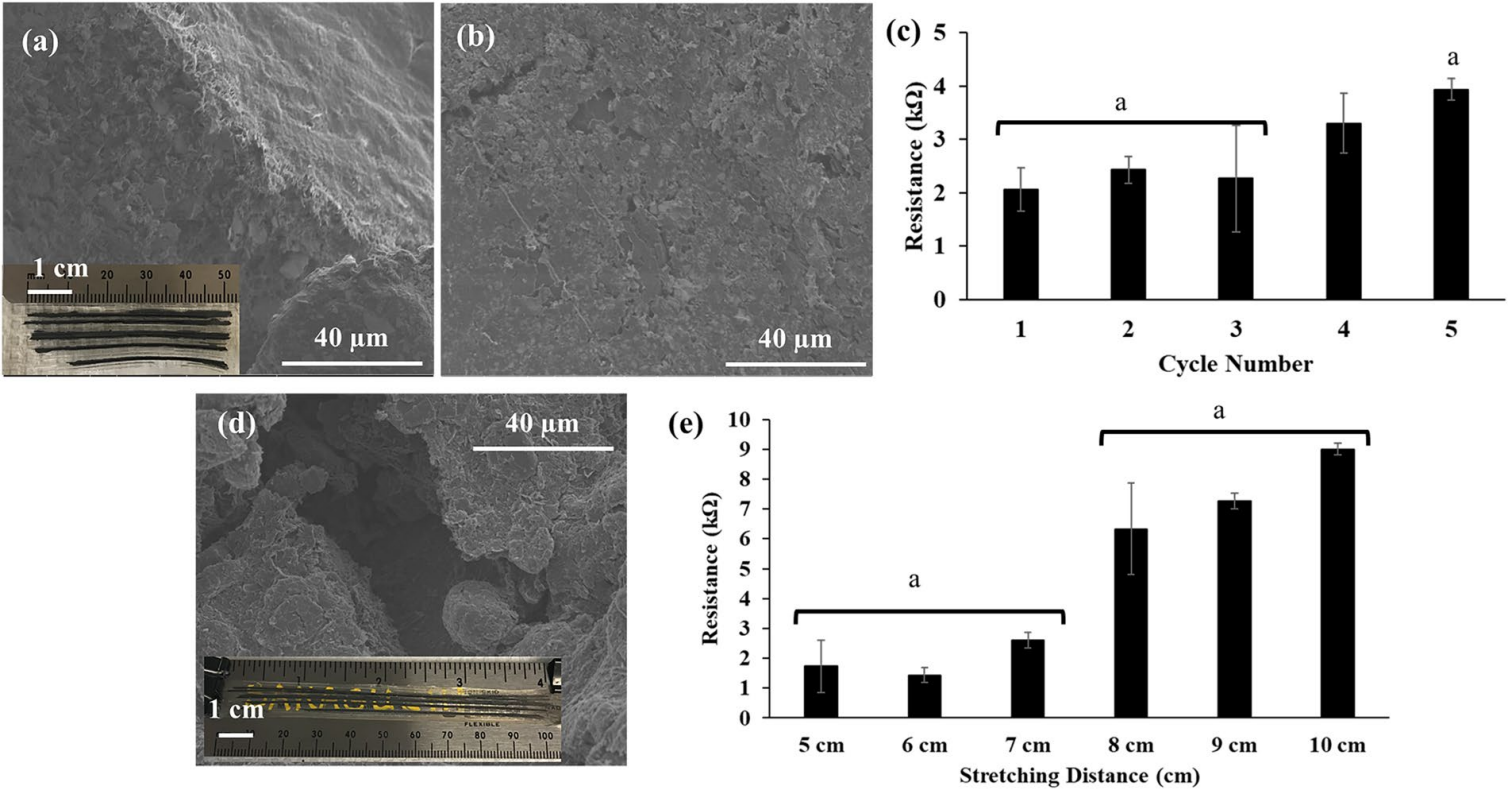
SEM images of graphene patterns transferred on WPI films (**a**) before (5 cm initial length) and (**b**) after 5 cycles of stretching test (3 times of 2 cm stretching and retraction was applied) (**c**) Change of the resistance after cyclic stretching test. (**d**) SEM images of graphene patterns transferred on WPI films after 10 cm of (the final stretched length) stretching. Stretching distance is 5 cm. (**e**) Change in the resistance as the stretching distance increases to the final distance of 10 cm. For (**c**,**e**) ANOVA analysis by Tukey’s method with a 95% confidence interval was conducted and the p value was found to be smaller than 0.05 for indicated letter “a” showing significant difference. (n = 3, and error bar represents one standard deviation from the average).

As an alternative to the flexible electronics, this novel method can also be used to fabricate stretchable electronics. For this purpose, we fabricated flexible and stretchable WPI films with conductive graphene patterns using polymer casting method. The initial length of WPI film with graphene patters was 5 cm (Fig. 6a). A cyclic stretching test (5 cycles: 3 times of 2 cm stretching and retraction) was applied to detect the changes in the graphene pattern structure and resistance (Fig. 6b,c). Figure 6b indicated that after cyclic stretching test there was no significant change in the graphene structure, which also resulted in the observation of stable resistance in the graphene patterns as illustrated in Fig. 6c. Then, the WPI film was stretched gradually (1 cm each time) up to 10 cm (Fig. 6d). Figure 6d,e show that after 5 cm of stretching, the graphene structure started to break its continuity due to the effect of extension, which in turn resulted in increased resistance and decreased conductivity (Fig. 6e). The patterns were able to maintain their resistance around 2 kΩ up to 2 cm stretching (final extension till 7 cm) after which the resistance increased up to 9 kΩ when the stretching reached to 5 cm (final extension till 10 cm). This is clearly due to the discontinuity of the graphene patterns upon stretching as indicated in SEM images. Nevertheless, this experiment indicated that it is possible to fabricate stretchable electronic circuits with polymer casting method.

These results clearly demonstrate that it is easy, fast, green and cost effective to fabricate graphene-based flexible and stretchable electronic devices on various biodegradable and biocompatible polymeric flexible substrates with proper 3D microstructural properties, small feature sizes and high-resolutions via the mentioned method. Therefore, the fabricated devices can be used in various biomedical and healthcare applications. Considering this, we conducted additional experiments to demonstrate the potential use of the developed devices with polymer casting method for controlling the stem cell differentiation.

In our previous studies, we showed precise control of the transdifferentiation of stem cells using mechanical, physical, topographical, chemical and electrical cues for peripheral nerve regeneration purposes^70–73^. Very recently, we have demonstrated the successful transdifferentiation of mesenchymal stem cells (MSCs) into Schwann cells (SCs) using electrical stimuli through ink-jet printed and laser annealed graphene circuits on flexible polyimide substrates^70^. Although the transdifferentiation was successful, the non-biodegradable and non-porous nature of polyimide substrate limits the potential translation of this strategy for surgical implantation and clinical applications related to nerve regeneration. Therefore, the fabrication of such graphene-based circuit on biodegradable, 3D microstructured and porous substrates could make the surgical and clinical application of this strategy possible^74^. Therefore, the *in situ*-*in vivo* precise control on MSCs differentiation, migration and fate commitment upon the surgical implantation could be possible^75^. Considering this, we developed the same device (graphene circuit ink-jet printed and laser annealed on polyimide substrate) on biodegradable, 3D microstructured and porous PLLA films using our graphene transfer via polymer casting approach as described above.

**Figure 7:**
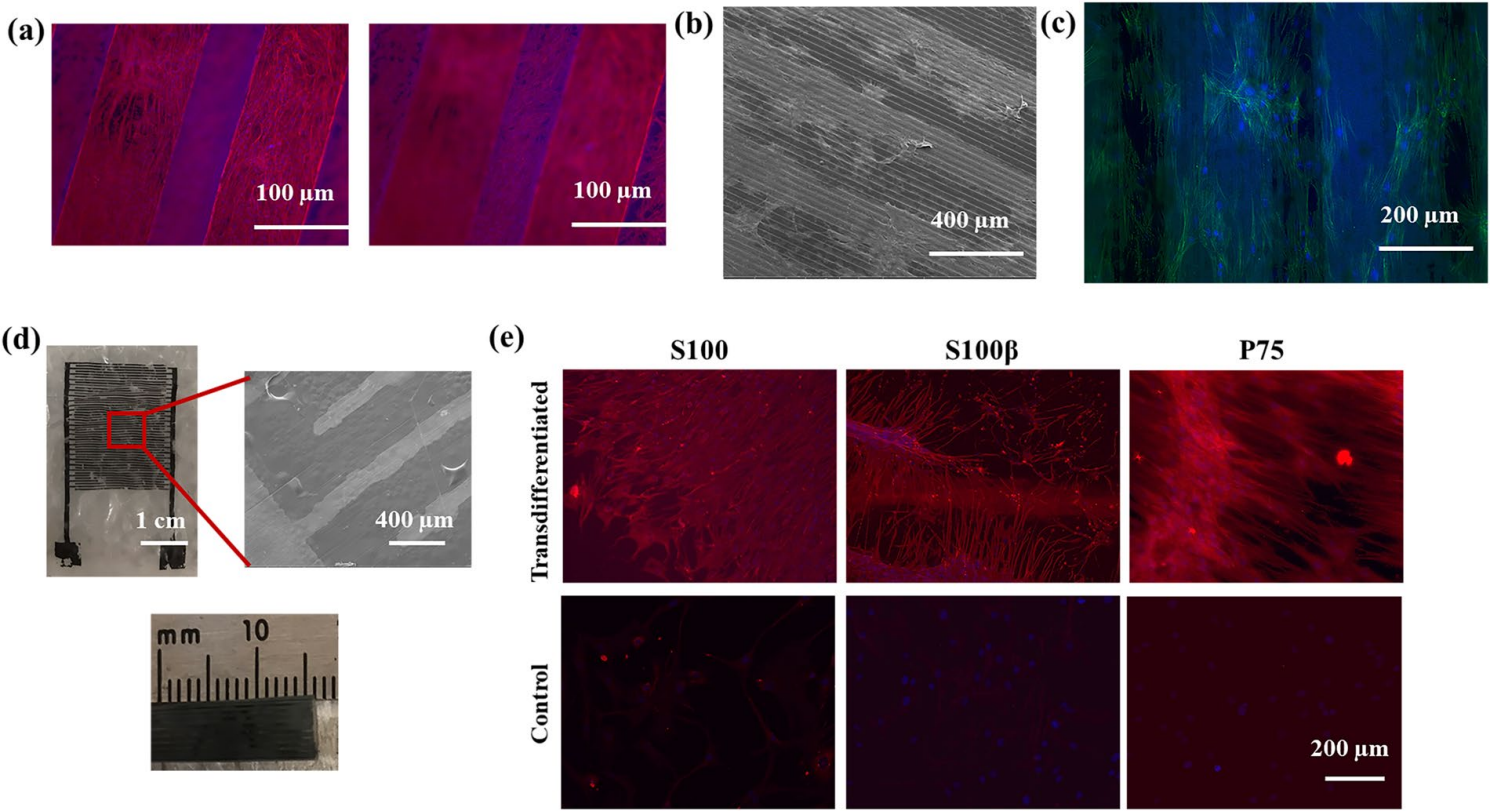
Demonstration of using fabricated devices for controlling the transdifferentiation of MSCs into SCs using electrical stimuli. (**a**) MSCs attaching and growing on the PLLA film and graphene patterns. (**b**,**c**) Directed growth of MSCs along the graphene patterns. (**d**) Graphene and PLLA-based device fabricated using microfluidic approach. (**e**) Immunocytochemistry staining of transdifferentiated MSCs with selected SCs markers.

We first investigated the attachment, growth and alignment of MSCs on the graphene patterns on the PLLA films. As demonstrated in Fig. 7a, MSCs grew both on the PLLA surface and graphene micropatterns. In addition, Fig. 7b,c also demonstrated the directed alignment of the MSCs along with the graphene patterns suggesting potential control on directional growth. Figure 7d shows the graphene circuit design on PLLA films fabricated using graphene transfer method. Figure 7d also illustrates that these circuits can be rolled into conduits as implants for specific peripheral nerve regeneration surgeries implying that they can potentially be used for other surgical or clinical applications. Following the attachment and growth of the MSCs on the devices, a specific electrical stimulus (100 mV at 50 Hz for 10 min per day for 10 days) was applied to the MSCs based on our previous study in order to differentiate them into SC-like phenotypes^70^. Our results showed that almost ~90% of the cells got immunolabelled with the selected SC markers suggesting the successful transdifferentiation (Fig. 7e). These results are in accordance with our previously published findings^70^ demonstrating the potential of the device and our new fabrication method. These results also confirm the use of this technology to control MSCs differentiation and further enables its potential application for surgical and clinical translation by developing biodegradable and biocompatible devices with our new polymer casting approach.

## Conclusions

This novel method enables the fabrication of flexible electronics based on graphene and various polymeric substrates with precise control of 3D microstructural and mechanical properties (such as film porosity, pore size, elasticity etc.). The flexibility in the polymeric material selection along with precise control on substrate properties, enables the potential use of the developed devices in biomedical applications or implantations. In addition, with this approach it is possible to obtain graphene patterns with high resolution, low feature size (as low as 5 µm; 15 µm width and 5 µm depth), high conductivity (sheet resistance of ~0.2 kΩ/sq) and high stability (maintains sheet resistance after 100 bending and 24 h washing cycles). Moreover, this is a novel, facile, versatile, scalable and cost-effective manufacturing approach that eliminates the need for expensive equipment (except for initial lithography to create small microchannel patterning for the molds), physical or chemical post-processing or complex transferring/stamping processes. Furthermore, this is an alternative approach to the conventionally used PDMS molding or Cu foil-based transfer methods. Overall, this promising method has the potential to pave the way for flexible and stretchable electronics fabrication, particularly for the biomedical applications including but not limited to brain-computer interfaces, robotics or lab/organ-on-a-chip.

## Methods

### Preparation of substrates with graphene patterns/films

The Teflon or Delrin substrates with small feature size micropatterns were prepared using computer numerical control (CNC) machine while the silicon wafer molds with small pattern features were prepared using photolithography only once. The pattern dimensions were varied between 5 to 400 µm in width and dept. Graphene nanoplatelet (Sigma Aldrich) solution, sonicated and thermally annealed (at 75 °C) prior to application (pre-annealing), was used to fill the micropatterns. The excess graphene on the substrate was removed and cleaned by sticking and peeling a commercially available scotch tape, which leaves the graphene in the patterns^46^. In another approach, a graphene pattern was ink-jet printed on a rigid or flexible polyimide substrate and thermally or laser annealed (to enhance the conductivity) to create graphene substrates^59,70,76^. In addition, graphene and silver nanowire films were grown on quartz and Cu foil substrates via conventional CVD method^23,77^.

### Polymer casting, film formation and transfer of graphene

**Figure 8:**
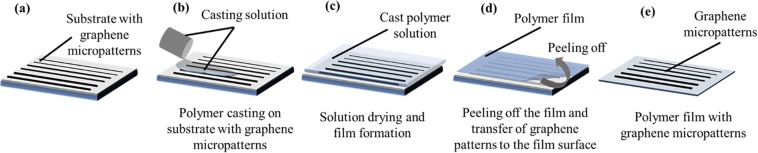
Graphene transfer via polymer casting. (**a**–**e**) Schematic representation of graphene transfer via polymer casting. (**a**) Substrates with graphene micropatterns. (**b**) Casting of polymeric film formulation on substrates with graphene-based micro-circuit patterns of various feature sizes. (**c**) Drying of casting solution and film formation. (**d**) Peeling off the polymeric films and transfer of graphene-based micropatterns from the rigid or flexible substrate to the film surface. (**e**) Polymeric film with graphene micropatterns on the surface.

Following formation of graphene patterns on the substrates using the techniques mentioned above, the polymer casting solutions were prepared at desired formulations and concentrations. The polymer solution (could be Poly-L-Lactic Acid (PLLA), Cellulose Acetate (CA), Gelatin (GEL), Poly Lactic-co-Glycolic Acid (PLGA) or Whey Protein Isolate (WPI)) was then cast on the substrate with graphene patterns/films and left for drying. The 3D microstructure, mechanical properties, porosity and pore size of the films can be adjusted via well-established phase inversion techniques or use of pore forming or plasticizer agents. Upon the film formation, the film was peeled off and graphene patterns were transferred from the substrates to flexible film surface (Fig. 8a–e). This polymer casting approach can be applied for both rigid and flexible graphene substrates.

### Characterization of the prepared devices

The stability of the graphene patterns on the polymer devices was tested through multiple washing, bending and peeling-off cycles. The conductivity of the devices was determined by building up a circuit and measuring resistance. The microstructure of graphene patterns and devices were characterized through scanning electron microscopy (SEM) (FEI Quanta 250 FE-SEM), x-ray photoelectron spectroscopy (XPS) (Amicus XPS) and Raman spectroscopy (Bruker FT-Raman Spectrometer) analysis. SEM samples were sputter coated with 2 nm iridium before the analysis and images were taken using secondary electron mode. Monochromatic Al Kα X-ray source (1486.6 eV) was used in XPS analysis with an electron take-off angle 45° from a normal sampling surface. Survey scans were collected from 10 eV to 1100 eV with a pass energy of 187.85 eV. Raman spectra were collected with a backscattering geometry, 1064 nm Nd:YAG laser and a spot size of about 1 mm.

### Use of graphene devices as biointerfaces

We investigated the potential use of the fabricated devices as biointerfaces to control the stem cell behavior. We used Brown Norway rat mesenchymal stem cell (MSCs), which were provided by Dr. Donald S. Sakaguchi in Genetics, Development and Cell Biology Department at Iowa State University.

The MSCs isolation procedure from Brown Norway rats was conducted in accordance with the NIH Guide for the Care and Use of Laboratory Animals guidelines and the principles in the “Guidelines for the Use of Animals in Neuroscience Research” presented by the Society for Neuroscience. All animal procedures had the approval of the Iowa State University Institutional Animal Care and Use Committee and were performed in accordance with committee guidelines.

MSCs were plated in T75 flasks in maintenance media (MM), consisting of α minimum essential medium (αMEM, Gibco BRL), 20% fetal bovine serum (FBS; Atlanta Biologicals), 4 mM l-glutamine (Gibco), and antibiotic–antimycotic (Invitrogen) and incubated at 37 °C and 5% CO_2_. MSCs were sub-cultured when they reached 80% confluency approximately every 2–3 days.

The ink-jet printed graphene patterns with finger dimensions of 400 µm finger width and 250 µm finger-to-finger spacing were transferred to the surface of PLLA film by applying polymer casting method and the obtained devices were used for electrical transdifferentiation of MSCs. 2 × 10^5^ cells were seeded on devices and electrical transdifferentiation into Schwan cell-like phenotypes (SCs) was conducted exactly as described in our previous work^70^. At the end of transdifferentiation, immunocytochemical analysis was performed on MSCs possessing SC-like phenotypes seeded on the device using selected SC markers, s100, s100β and p75 as described previously^70^.

## Data Availability

The datasets generated during the current study are available from the corresponding author on reasonable request.
